# Stakeholder views on the installation and use of mile long tracks in community parks aimed at increasing physical activity in low-income minority areas: A qualitative evaluation in Birmingham UK.

**DOI:** 10.1016/j.puhip.2026.100758

**Published:** 2026-02-28

**Authors:** Ameeta Retzer, Peymané Adab, Nina Sa Fischer, Emma Frew, Janet Jones, Hisham Y. Makahleh, Anwesa Manna, Miranda Pallan, Irina Pokhilenko, Gavin Rudge, Rhona Duff, Mohammud Ibrahim Subdurally-Plon, Kate Jolly

**Affiliations:** a Department of Applied Health Sciences, College of Medicine and Health, University of Birmingham, Edgbaston, Birmingham, B15 2TT, UK; b Adult Social Care and Health Directorate, Birmingham City Council, PO Box 16616, Birmingham B2 2HN, UK

**Keywords:** Low-income neighbourhoods, Ethnic diversity, Communities, Physical activity, Qualitative

## Abstract

**Objectives:**

Physical activity (PA) is essential for preventing and managing chronic diseases and improving mental health. Despite national guidelines recommending regular PA, significant disparities exist across demographic groups and regions in the UK, with Birmingham and the West Midlands reporting some of the lowest activity levels. We aimed to explore the development, implementation, and perceived value of Every Step Matters (ESM) tracks—mile-long walking routes installed in eight Birmingham parks situated in neighbourhoods with high deprivation and ethnic diversity.

**Study design:**

Qualitative evaluation.

**Methods:**

Data were collected from 35 participants through ten in-depth interviews and four focus groups, including community members, track users, and stakeholders from the Birmingham City Council (BCC) Public Health team and a physical activity charity based in the West Midlands who work with communities from a range of socio-economic backgrounds and ethnicities in urban areas. Rapid qualitative analysis was undertaken.

**Results:**

Seven themes were identified: (1) use of the tracks, (2) promoting use, (3) increasing appeal, (4) perceived benefits, (5) barriers, (6) sustainability, and (7) interactions between the Public Health team and the PA charity partner. While the tracks were viewed as inclusive, low-cost, and beneficial for health and community cohesion, barriers such as safety concerns, low motivation, and poor maintenance were identified. Recommendations include improving awareness, addressing safety, promoting group activities, and ensuring long-term sustainability.

**Conclusions:**

The ESM tracks show promise as a community-based intervention to promote PA in disadvantaged areas. However, sustained impact requires strategic planning, community ownership, and targeted support. Further research is needed to explore adherence, guided versus non-guided use, and the role of social determinants in PA engagement.

1 **Plain English summary**

1.1 **Why walking matters**

Being active helps you stay healthy and feel happy. But lots of people in the UK—especially in Birmingham and the West Midlands—do not get enough exercise. To help, the *Every Step Matters* project built mile-long walking tracks in eight Birmingham parks. These parks are in areas where people have fewer opportunities and come from different backgrounds.

Researchers wanted to find out how these tracks were made, how people use them, and what they think. They talked to thirty-five people, including residents, track users, and staff from Birmingham City Council and a charity that promotes exercise.

1.2 **What they found**

• People liked the tracks because they are free, easy to use, and good for health and community spirit.
• Problems included safety worries, low motivation, and poor maintenance.

1.3 **Ideas to make it better**

• Tell more people about the tracks.
• Make them safer.
• Organise group walks.
• Keep them in good condition.

The tracks could help people in these areas be more active. But for this to work long-term, projects need good planning, community help, and extra support. More research is needed to see if people keep using the tracks, if walking with a guide works better than walking alone, and how friends and family affect exercise.

Building and looking after tracks takes time, money, and effort. Collaborating with the community from the start is important. Promoting the tracks in ways that fit the local area and organising walking groups could help more people join in—but these need extra resources.

2 **What this study adds**

• This study has highlighted the insufficient research on the practicalities, time, and funding needed to develop, install, promote, and maintain effective physical activity facilities such as mile tracks in public parks within neighbourhoods with high deprivation and ethnic diversity.
• Several barriers to the use the tracks were identified, the main concern being personal safety and security.
• This study provides insights into park users and residents' views about the installation and use of mile long tracks in neighbourhoods with high deprivation and ethnic diversity.

3 **Implications for policy and practice**

• From inception, it is crucial for everyone involved in similar projects, including potential users, to have a good working relationship. This helps avoid misunderstandings about the project and the roles of those involved.
• To increase and maintain usage, it is recommended to promote the tracks with a targeted and tailored approach, ensuring all residents are aware of the installation and its purpose. Different community groups may need different promotional strategies.
• Organising walking groups could boost track usage and address safety and security concerns, though it may incur additional costs.

## 4 Introduction

Physical activity (PA) offers significant physical and mental health benefits, helping prevent and manage chronic conditions like cancer, heart disease, diabetes and depression [[1], [2], [3]] and it is a key risk factor for mortality in high-income countries [4]. Economically, increased PA reduces the need for healthcare, thereby reducing healthcare costs, and boosts productivity [5]. The UK government prioritises health-promoting activities, especially PA, for public health investment [6,7].

The UK Chief Medical Officer's (CMO) Guidelines recommend that adults do 150 min of moderate or 75 min of vigorous PA weekly, plus strength exercises twice a week, and reduce sitting time [8]. However, 34% of men and 42% of women do not meet these guidelines [4]. PA levels also decline with age [9]. There are further inequalities in PA participation by ethnicity and disability status. Active Lives Survey data show lower PA levels among Asian, Black, and Chinese ethnicities, when compared to white British ethnicity [10,11] and people with disabilities or long-term conditions are twice as likely to be inactive, with 43% of disabled people and 21% of non-disabled adults being inactive. Additionally, the survey reports 35.3% of adults from the most deprived areas are inactive and the proportions of children and young people who are active is lowest amongst those from the least affluent families [12]. Research in the US has shown that low-income minority neighbourhoods have fewer recreational facilities than wealthier, predominantly white communities [[13], [14], [15]].

Birmingham has a population of 1.1 million and is the most densely populated area in the West Midlands. The average age of the population is 34 years of age with 40% of these under the age of 25 compared an average age of 40 across England. Birmingham is one of the most deprived areas in the UK with 44% of the population living in 10% of the most deprived areas in England [16,17].

There are also regional variations in PA; in the UK, the West Midlands is one of the least active regions. Survey data collected during the pandemic from Birmingham, a large city in the West Midlands region, showed that 49.8% of adult respondents reported becoming less active since the start of COVID-19 compared to 37% nationally [18]. A similar pattern is evident in children and young people; in 2020-21, 44.6% of children and young people in school years 1-11 (aged 5-16) in England met the CMOs’ guidelines of taking part in sport and PA for an average of 60 min or more every day. Regional rates were lowest in the West Midlands (42.0%) [19]. Research shows that parents can be PA role models and gate-keepers to opportunities for their children to be active [20], therefore, increasing PA in adults is also important for PA in children.

### 4.1 Every step matters (ESM) track installations in Birmingham, UK

The World Health Organisation recommend that PA should be integrated into where people live, work and play [21], this recommendation underpinned the development of the ESM tracks in Birmingham, UK. ESM tracks are painted mile-long tracks intended to be used daily. These were installed in eight parks located within Birmingham wards that have high deprivation, high ethnic diversity, and low levels of PA.

This intervention is part of the Birmingham City of Nature Plan [22]. which has the aspiration of opening up equal access to the natural environment for all residents of Birmingham. The tracks were installed by the charity partner, who were also commissioned to engage with local communities and target groups within the eight localities by promoting the tracks through social media, flyers and organising events in the parks to encourage the use of the ESM tracks for leisure time physical activity. The ESM programme later evolved to incorporate guided walks for which they recruited volunteer walk leaders to ensure the safety of the walkers, manage the pace and welcome new participants.

### 4.2 Aims of this study

The aims of this study were: 1) to evaluate the development and installation of the ESM tracks, 2) to identify the barriers and facilitators to using the ESM tracks and 3) to ascertain the perceived value of having access to the ESM tracks.

## 5 Methods

This study obtained ethical approval from the University of Birmingham Research Ethics Committee (reference number: ERN_1041-Oct2023). All participants provided informed consent prior to taking part in either an interview or focus group.

Below is a brief synopsis of the methods but due to the heterogeneous approach to recruitment depending on the specific group of participants a detailed summary of the methods is provided in appendix 1 and the COREQ checklist is in appendix 2.

### 5.1 Data collection

We conducted in-depth interviews with employees of the charity partner (including walk leaders) with experience of working with BCC public health team on the development and installation of the ESM tracks to explore elements that worked well and areas that could be improved. We also conducted in-depth interviews with members of the local communities who have used the ESM tracks to explore the behavioural components of usage of ESM tracks, including previous exercise regimen, reasons for changes, and current use. Focus group discussions were undertaken with non-track users to identify the barriers and enabling factors for use of the ESM tracks. Additionally, all participants were asked about their perceived value of having access to and using the ESM tracks. All track users and non-users were recruited in the parks or from the local vicinity, approximately within one mile of the park. Interviews with track users were conducted in-person at the point of recruitment or if preferred at a later date via telephone or video-conferencing software. Focus groups with non-users took place in previously hired venues within ½ mile of the park. *Walk leader*s were identified through the charity partner and telephone interviews arranged. Interview and focus group topic guides were piloted with a lived experience group and iteratively amended as data collection progressed. All interviews and focus groups were audio-recorded and transcribed verbatim.

### 5.2 Analyses

Data collected were analysed using Rapid Research Evaluation and Appraisal Labs (RREAL) sheets, a rapid qualitative analysis [23] tool. A team-based approach to coding adapted from Geisen et al. [24] was used. Our team consisted of an experienced qualitative researcher, two junior coders and a project lead also experienced in qualitative research (see Appendix 1 for experience of team members). The experienced researcher developed an initial framework informed by the research aims and provided training and ongoing support to the junior coders. The coding was applied to one transcript and discussed within the team as part of the training. Following this each junior member of the team, coded transcripts individually and then met with one of the experienced researchers to discuss the coding. All members of the team then met weekly to discuss and review progress. A flexible and iterative approach was used to continually develop and refine the coding frame, allowing for the interpretation of novel themes. Additional codes were developed and integrated as analysis progressed. Disagreements were resolved through discussion. Illustrative quotes were identified from the RREAL sheets and drawn from the audio-recordings or transcripts.

## 6 Results

Thirty-five individuals participated: 25 non-users in focus groups (facilitated by …), eight ESM track users were interviewed (by ….) and two from the charity partner and Birmingham City Council Public Health team (…). Table 1 shows the demographics of track users and non-users. Interviews lasted between 10 and 40 min (mean 16 min), focus group discussions lasted between 50 and 90 min (mean 62 min).

**Table 1:** Public Participant demographics.

	Demographics	Non-users (n = 25)	Users (n = 8)	Total (n = 33)
Age				
	18-45	10 (40%)	4 (50%)	14 (42%)
	46-65	6 (24%)	1 (13%)	7 (21%)
	66 and above	9 (36%)	3 (38%)	12 (36%)
Gender				
	Woman	19 (76%)	3 (38%)	22 (67%)
	Man	6 (24%)	5 (63%)	11 (33%)
Ethnicity				
	British/White groups	3 (12%)	7 (88%)	10 (30%)
	South Asian (Indian, Pakistani, Bangladeshi)	8 (32%)	1 (13%)	9 (27%)
	African/Caribbean	10 (40%)	0 (0%)	10 (30%)
	Mixed	1 (4%)	0 (0%)	1 (3%)
	Not indicated	3 (12%)	0 (0%)	3 (9%)

Seven themes were identified from the qualitative data collection [1]: Use of the tracks [2], Promoting use of the tracks [3], Increasing appeal of the tracks [4], Perceived benefits of the tracks [5], Barriers to using the tracks [6], Sustainability [7], Interactions between Public Health department and charity partner. Table 2, supporting quotes are provided in Table 3.

**Table 2:** Summary of themes and subthemes.

Theme	Sub-theme
Use of the tracks	Guided walks
	Other forms of activity
	Children
Promoting use of the tracks	Methods of promotion
	Signposting
Increasing the appeal of the tracks	Safety
	Maintenance of the overall park
	Gamification
	Walking groups
Perceived benefits of the tracks	Low-cost and inclusive
	Convenience of the location
	Social connections
	Physical and mental health
Barriers to using the tracks	Safety concerns
	Personal motivation
	Cost of living/cost benefits
	Comparison to other forms of exercise
Sustainability	Low participation
	Long-term financial viability
Interactions between public health team and charity partner	Planning
	Communication
	Priorities and practicalities
	Reflections

**Table 3:** Supporting quotes.

Quote No.	Theme and quote
	**Use of the tracks**
1.	*“Parts of the routes will always pass the playground area but we've yet to see young people utilising the routes.” (Charity partner_001)*
2.	*“We sometimes play a game where Mummy will choose a red one to go and then we will go on the yellow. We have little races on it, and she tries to balance on one of the colours. So, we do play games*.*” (User_004)*
	**Promoting use of the tracks**
3.	*“Commissioning Legacy WM worked well because they have the community connections, infrastructure and volunteers and are best placed to be most effective at engaging the community. Had the council implemented the tracks alone then people would have been quite sceptical about it.” (Public health team_001)*
4.	*“How would anybody know that? Because there's nothing. There's no sign or anything*.*” (NonUser_001)*
	**Increasing appeal of the tracks**
5.	*“The main thing with people is to create an environment where they don't feel judged and feel comfortable. Sometimes people feel embarrassed and judged while exercising in public. If there is a group where all the people doing the same will definitely give them confidence*.*” (NonUser_001)*
6.	*“There's a group of us who used to walk and all of a sudden one person dropped out and then the next one dropped out, then nobody was doing it*.*” (NonUser_FG2)*
	**Perceived benefits of the tracks**
7.	*“Do it because it will save you money. So absolutely that's an appealing aspect*.*” (NonUser_FG1)*
8.	*“That's a good way to calculate steps and distance without having to have technology” (User_002)*
9.	*She (friend) feels good about coming to the park for a run. It is cost-effective, and the road connect the destination and her home.* (User_002)
10.	*“Residents are making new acquaintances and friends, and some parks now have a WhatsApp group so everyone can keep in touch.” (Charity partner_001)*
11.	*“During lockdown, for instance, it was very good for the mental. I did not feel isolated at all, because I just came here, walk, breathe.” (User_003)*
12.	*“They found that to come out to the park on their own, it's them that escape from whatever issue they're suffering, I've actually realised it's a place of therapy for them.” (Walk leader_001)*
	**Barriers to using the tracks**
13.	*“I can't recall any time (during the day) feeling unsafe in the park because I was walking around, and other people were in the park walking as well*.*” (NonUser_FG3)*
14.	*“It is not human behaviour that puts me off the park, it is dogs” (NonUser_FG3)*
15.	*“I am trying to incorporate a gym routine simply because, like in the winter months is so much harder to be motivated and go outside*.*” (NonUser_FG3)*
16.	*Distance. If the park is far from their (friends) house, they would not like to drive to get to the park.* (Non-user_FG4)
17.	*“They can count exactly how much they are walking. Rather than using this line, people can use phones to track miles. There are loads of fitness apps in the market*.*” (User_005)*
	**Sustainability**
18.	*“I'd have to know about how much has been invested because I don't know if it would be more useful spent elsewhere. There's so much that needs doing around this area and a lot in Birmingham*.*” (User_004)*
	**Interactions between Public Health Team of BCC and charity partner**
19.	*“More detailed information and background research for future projects could help save time and reduce problems” (Charity partner_001)*
20.	*“I thought they were so disconnected. It's like the officers managing it didn't appreciate the challenges that we were facing, and for them it was like we need to get it done quickly.” (Charity partner_002)*
21.	*“There just seemed to be a bit of miscommunication between us.” (Charity partner_002)*
22.	*“I think commissioning the local organisation is the thing that's worked well …. They've got the relationships, infrastructure and you know volunteers.” (Public health team_001)*

### 6.1 Use of the tracks

#### 6.1.1 Guided walks

Weekly walking groups were set up in some parks which were perceived by the charity partner to be well attended, although some participants reported decreasing numbers (Quote 6).

#### 6.1.2 Other forms of exercise

It was observed that the tracks were sometimes used for other exercises such as cycling, sprinting and drill exercises.

#### 6.1.3 Children

Despite purposely routing tracks past playgrounds, children's uptake was lower than expected. (Quote 1) However, one participant used the colourful markings as a game with their child. (Quote 2).

### 6.2 Promoting use of the tracks

#### 6.2.1 Methods of promotion

Engagement with local community-based organisations and existing walking groups was a key step in the development and promotion of the tracks. (Quote 3) the charity partner undertook a range of activities to promote the tracks. These included the use of social media, distribution of flyers, listing the tracks on a local sport activity finder (Active Birmingham) and local engagement events with emphasis on cultural sensitivity. However, most non-users were unaware of the promotional efforts and those who saw the promotions felt they were ineffective.

#### 6.2.2 Signposting

Both user and non-user participants felt that the signposting was poor and park users who had not seen the promotional materials were confused about the tracks' purpose and found the limited signage near the track unhelpful. Overall participants felt that more information could be included such as the length of the track, number of calories burned and directions to the start of the track. (Quote 4).

### 6.3 Increasing appeal of the tracks

#### 6.3.1 Safety

Personal safety and security whilst using the tracks was a priority for the interviewees who described how improving perceptions of personal safety will encourage people to use the park.

#### 6.3.2 Maintenance of the overall park

All groups, except the non-users reported that good general maintenance of the park including well-kept gardens, play areas, and litter bins, can encourage more people to use the park and the tracks.

#### 6.3.3 Gamification

“Gamification” of the tracks was suggested by the charity partner, including motivational signs *i.e. “Keep going, still 0.5 miles to complete a mile.”* and incentives like rewards for completing tracks. Although how these rewards would be delivered was unclear.

#### 6.3.4 Walking groups

Interviewees agreed that walking groups boost confidence and reduce judgment because everyone is doing the same thing. (Quote 5) However, some were concerned about the sustainability of walking groups, especially informally organised ones, due to dropouts. (Quote 6).

### 6.4 Perceived benefits of the tracks

#### 6.4.1 Low cost and inclusive

The main benefit identified by the participants was that the tracks offer a low-cost inclusive activity that does not require any specialist equipment or ability to undertake. (Quotes 7 and 8).

#### 6.4.2 Convenience of the location

Additionally, the convenience of the location of the parks was appreciated by users who described how the short distance from their homes to the park encouraged them to go to the park frequently. (Quote 9).

#### 6.4.3 Social connections

The tracks were seen as good for families because they are colourful, bright attractive and a clever way for children to do exercise and get out of their houses into the fresh air. Although use of the tracks by young people was perceived to be minimal. (Quote 2) Participants also explained how the tracks can foster a sense of community by making new friendship groups. (Quote 10).

#### 6.4.4 Physical and mental health

The potential benefits to mental and physical health were highlighted; Walking outside in the fresh air can be good to combat isolation and low self-esteem. The benefits in terms of fitness and weight management particularly for older people were also recognised. (Quotes 11 and 12).

### 6.5 Barriers to using the tracks

#### 6.5.1 Safety concerns

Personal safety was of most concern to non-users because of high levels of anti-social behaviour in their respective areas, although this was not unanimous. (Quote 13) Overall, the users did not have such concerns as they believed the areas to be reasonably safe. BCC and the charity partner highlighted that more walkers on the tracks may reduce anti-social behaviour.

Limited lighting deterred non-users who prefer to exercise in daylight but were unable to due to work commitments, and track users confirmed that they tended to go during daylight. It was acknowledged that parks are public areas and dogs not on a leash caused anxiety to some non-users making them feel unsafe. (Quote 14).

The visibility of the tracks was reported as poor, for example, some lines were painted over fallen leaves rather than sweeping them away prior to painting. Additionally, failing to remove fallen leaves could make the tracks slippery to walk on.

#### 6.5.2 Personal motivation

Self-motivation was key for both users and non-users. Adverse weather made it harder to have the motivation to get out of the house. (Quote 15) The boredom of walking along the same line every time put one non-user off using the tracks and having to travel to the park was an obstacle mentioned by one of the non-users (Quote 16).

#### 6.5.3 Cost of living/cost benefits

In addition, some non-users explained how they had other priorities such as meeting the cost of living rather than exercising.

#### 6.5.4 Comparison to other forms of exercise

Those involved in alternative exercise routines, including paid ones, may choose not to use the tracks. Some participants indicated that activity trackers provide more accurate and comprehensive data irrespective of the user's location, thereby reducing the need to use the tracks. (Quote 17).

### 6.6 Sustainability

#### 6.6.1 Low participation

There was concern about the sustainability of the tracks. One user commented that there is no benefit in using the tracks as one could walk around the park itself, and one said they had never seen anyone using the track.

#### 6.6.2 Long-term financial viability

It was recognised by many that the provision of walk leaders would require funds as would the general ongoing maintenance of the tracks and suggested the need to invest in the cleanliness and safety of parks more generally. Community ownership was suggested as it would make the tracks independent of the council. The need to invest in the basic wellbeing and health of the population rather than the mile tracks specifically was raised by some. (Quote 18).

### 6.7 Interactions between public health team and charity partner

#### 6.7.1 Planning

The preparation and installation of the tracks took much longer than expected. Participants described how in future projects, a realistic timescale with detailed information and background research should be scheduled along with an appropriate budget and resources. (Quote 19).

#### 6.7.2 Communication

Overall, both partners reported having a good working relationship. (Quote 20) However, at times communication between the teams was poor. (Quote 21) There seemed to be a misunderstanding on the interpretation of the tender to install the tracks (Quote 22) In addition, frequent changes of staff in the public health team led to a lack of continuity.

#### 6.7.3 Priorities and practicality

Both parties had differing priorities for example the public health team felt it was necessary to install tracks in areas of high anti-social behaviour and subsequently tackle the behaviour in partnership with the Violence Reduction Team. However, safety and security of employees was paramount to the charity partner, and this meant that contractors needed to pair up for safety. The charity partner was unable to recruit walk leaders in those areas and therefore needed to use their own volunteers/staff impacting on costs and resources.

#### 6.7.4 Reflections

A debrief between both parties was recommended by the Public Health team to discuss and identify where relationships could be improved and how future projects should be conducted.

## 7 Discussion

Park usage varies significantly by race, ethnicity, gender, age, and community income level, with access to green spaces often limited in lower-income areas [25,26]. To address these disparities, the Public Health team prioritized track placement in socioeconomically disadvantaged neighbourhoods, while the charity partner implemented culturally sensitive engagement strategies, consistent with current evidence [27]. This study evaluated the implementation and utilization of these tracks.

Participants highlighted several perceived benefits of the tracks, including the convenience of park location, improvements in physical health, and the positive impact of outdoor walking on mental health and overall well-being. These observations are consistent with existing evidence regarding the psychological benefits of walking, particularly in nature-based environments [28]. The advantages of guided walks are well documented, demonstrating safety, high adherence, and broad health benefits. However, participation in outdoor walking groups tends to be more common among socioeconomically advantaged populations, and current research rarely reports findings by socio-demographic characteristics or addresses underlying social determinants of health. To promote track usage in socioeconomically disadvantaged areas and strengthen social connections, the charity partner established organised walking groups. These initiatives aimed to foster community engagement and align with evidence suggesting strong adherence to group-based walking programs [29,30]. Contrary to this evidence, participants reported a decline in regular group attendance over time.

The primary barrier to using the mile tracks, as identified by non-users, was a perceived lack of safety within the park, attributed to concerns about anti-social behaviour. Existing literature suggests that such concerns are often linked to wider neighbourhood issues such as crime, homelessness, and drug-related activities rather than to the park itself. Conversely, current users regarded the parks as safe and expressed satisfaction with their use. This perception aligns with findings from the RAND study conducted in urban areas in the USA, which reported that 88% of respondents considered their local park to be safe [25].

The observation of the non-users that the marketing strategy employed was ineffective contrasts with prior evidence. For instance, Cohen et al. documented a 62% increase in park usage following the introduction of promotional materials such as posters, signs, and banners [25]. Furthermore, the long-term sustainability of the tracks appears to depend on factors including ongoing maintenance, the visibility and clarity of signage, and broader determinants of park use (e.g., weather conditions and perceived personal safety). These elements necessitate oversight by a designated organisation or institution; the current project did not incorporate a plan for the maintenance of the tracks.

Our findings are similar to those reported by Droomers et al. in the Netherlands who found no difference in walking for leisure at least once per week for the residents living in 24 of the most deprived neighbourhoods following improvements to local parks [31]. To encourage people to increase their PA levels the Centres for Disease Control in the US recommends a combined approach of enhancing access to PA places and providing information to encourage use of these places [32,33]. However, further research is needed on the factors affecting the usage of mile tracks such as those in ESM, comparing guided versus non-guided walks, and whether formal or informal organisation impacts adherence and sustainability [34]. In addition, how to address concerns regarding personal safety were not explored in detail in the current study. When considering the effectiveness of walking interventions in areas with relative socio-economic disadvantage and deprivation, this is important and should be explored further in future research.

### 7.1 Recommendations for future projects

The findings from this research led to the identification of five key areas of recommendation for those embarking on similar projects in the future [1]: promoting use of the tracks [2], sustainability and ownership [3], engaging holistically [4], personal safety and inequalities, and [5] transparency (Table 4).

**Table 4:** Recommendations for future installation of mile tracks in public parks.

**Promoting use of the tracks**
Using a wide range of promotional formats and linking them with other events and services in the local areas may improve awareness in the target population.
Explore the game-ifying of the tracks by using applications such as Active Birmingham.
Greater usage of physical signage could be employed to make the track itself more interactive i.e. having motivating slogans or indicating the distance walked.
**Sustainability and ownership**
For long-term sustainability, stakeholders willing to take ownership and responsibility for the tracks should be identified early in the process.
There were cases where the tracks were combined with pre-existing walking groups – this could enable longer term sustainability and facilitate the newer walking groups to become established
**Engaging holistically**
Consider target users' wider needs and how these can be met through engagement with the tracks. For example, through integrating health checks or information provision as part of the initiative may help to improve engagement.
**Personal safety and inequalities**
When planning for implementing future initiatives, ensure there is sufficient resource to enable staff/volunteers to work in pairs to promote personal safety.
When promoting awareness of the tracks to public and local communities, outline efforts to address antisocial behaviours and poor perceptions of personal safety including collaborations with violence reduction unit and local police.
When planning routes in various sites, consider whether areas are well-lit and if there are emergency call boxes nearby and if these can be part of the track routes.
**Transparency**
Where possible and appropriate, share information with the public about how funding decisions are made and the conditions for continuing worthwhile programmes.

### 7.2 Strengths and limitations

A strength of the study is that recruitment and data collection methods were planned to maximise inclusion, such as undertaking in-person canvassing in communities, offering a range of formats for providing consent and participating, and undertaking data collection in-person in community-based venues within one-mile of the tracks. The research team consisted of members fluent in widely used local languages, including Urdu and Punjabi, although they were not required. Efforts were made to develop and maintain links with local community networks to generate trust.

A limitation is that due to the short timescale and limited funding we were unable to produce the study literature in other languages, to overcome this we ensured that a research assistant was available to verbally translate but as stated above this was not requited. The charity partner could count participants in guided walks but could not quantify independent track users. Future research would benefit from alternate methods and new technologies to monitor footfall. A further limitation is that the ESM intervention and evaluation coincided with Birmingham City Council's Section 114 notice in September 2023, indicating the council was in financial difficulty. Once a notice is issued, the authority cannot incur new spending without permission from the finance officer [35]. While this did not impact on the delivery of the ESM programme or its evaluation, it was highly publicised and may have had an impact on the views expressed by those taking part in the evaluation. Some participants cited this when sharing their views of the tracks. Further, in Autumn 2023, there was a highly publicised national ban on certain dog breeds. This may also have affected the views shared by participants during the evaluation.

### 7.3 Conclusions

We undertook an evaluation of mile tracks installed in community parks in low-income minority areas to understand the barriers and facilitators to their installation and their use by residents. Our findings have highlighted areas to be considered when implementing similar projects in the future and suggest areas for future research such as investigating how effective walking interventions are on those living in areas of high deprivation and socio-economic disadvantage.

## Ethical statement

This research was reviewed and approved by the University of Birmingham Research Ethics Committee (Ref number: ERN_1041-Oct2023).

## Funding

This research was funded by Birmingham City Council (Ref: BCC20210000237A) with support from 10.13039/501100022244NIHR Applied Research Collaboration West Midlands.

## Declaration of competing interest

The authors declare that they have no known competing financial interests or personal relationships that could have appeared to influence the work reported in this paper.
