# Supplementary material for: Stakeholder views on the installation and use of mile long tracks in community parks aimed at increasing physical activity in low-income minority areas: A qualitative evaluation in Birmingham UK
Source: Public Health Pract (Oxf). 2026 Feb 28;11:100758. doi: 10.1016/j.puhip.2026.100758 (PMC12969091; doi:10.1016/j.puhip.2026.100758)
Supplement: Multimedia component 1 [file mmc1.docx]

**Appendix 1 – Every step matters evaluation methods**

***Researcher information***

Interviews and focus groups were conducted by an experienced qualitative research fellow or two research assistants (one male and one female), with master’s degrees, supervised by the project lead (experienced in qualitative methods) and the experienced researcher. The research assistants were fluent in Urdu and/or Punjabi. One of the research assistants was acquainted with three focus group participants, and to mitigate any bias the experienced researcher facilitated this focus group. All interviews and focus groups were audio-recorded, additionally the researchers took notes. There were no repeat interviews or focus groups and only the researchers and participants were present. Participants were not given the opportunity to read or comment on the transcript of their interview or focus group.

***Participant consent***

Consent was taken by the researcher. Consent form completion was offered in verbal, hardcopy, electronic and online formats. These options were intended to minimise burden on participants and accommodate a range of circumstances and preferences. Consent was taken in advance of the interview/focus group. Verbal consent was taken over the phone or via video conferencing software and was audio-recorded. Hard-copy, electronic and online consent forms were completed and returned to the researcher. Participants were informed of their right to decline participation, that participation was entirely voluntary and that all responses were completely confidential. Participants were also informed that they could withdraw from the study at any time before or during the interview/focus group discussion.

***Staff interviews – recruitment and data collection***

Participants were over the age of 18 and had experience in the development and installation of the Daily Mile tracks. They were also aware of the purpose of this evaluation.

*Recruitment*

Staff were recruited via email with standard text containing instructions to contact the researcher organising and undertaking the interviews. The email also included an attachment containing the Participant Information Sheet and Consent Form. The researcher and participant identified a mutually convenient time for the interview. A flexible approach was taken to facilitate participation. Recruitment continued until data saturation was reached.

*Data collection*

Interviews were chosen as the mode of data collection to enable in-depth exploration of individual experiences. Interviews took place remotely using secure video-conferencing software or by telephone. A topic guide (See below) was used to explore experiences of engagement with residents in developing approaches for encouraging use of the tracks, as well as barriers and facilitators identified. In addition, we explored views around additional resources that could facilitate further engagement and motivation for using tracks.

***Non-users - recruitment and data collection***

*Sample*

Participants were members of communities who did not use the Daily Mile tracks installed in their local park. Participants were over the age of 18 and based in Birmingham. Recruitment continued until no new themes were identified.

Five parks were purposively selected to capture maximum demographic diversity in terms of ethnicity, culture, and experience: We also aimed to recruit participants of different age and sex, as well as those with disabilities.

*Recruitment*

Participants were recruited via:

- Canvasing at community spaces proximate to the Daily Mile tracks
- Canvasing in the parks where a Daily Mile track is installed.
- Snowball recruitment through those contacted as part of the research.

*Data collection*

Focus group discussions were selected as the mode of data collection to maximise on group dynamics and to enable wider discussion of community issues relating to use of Daily Mile tracks. The focus groups were conducted in-person. Telephone or in-person interviews were available for those who were unable to make a focus group. In preparation for focus group sessions to take place in-person, community spaces such as religious buildings and community centres proximate to the tracks were identified and room bookings secured. Sessions lasted between 50 and 90 minutes. A topic guide (see below) was used to explore whether and to what extent previously identified or additional barriers may have prevented them from using the tracks and to understand what practical, psychological or social support could facilitate more widespread use of the tracks.

*Compensation*

Participants received a £15 shopping voucher following completion of the focus group discussion as a token of thanks.

***Track users - recruitment and data collection***

*Sample*

Participants were members of communities who have used the Daily Mile tracks installed in their local park. Participants were over the age of 18 and based in Birmingham. Recruitment continued until data saturation was reached.

As above the same five locations were purposively selected to capture maximum demographic diversity in terms of ethnicity, culture, and experience.

*Recruitment*

Participants were recruited via:

- Canvasing at the Daily Mile tracks
- Snowball recruitment through those contacted as part of the research.

The researcher and participant identified a mutually convenient time for the interview. A flexible approach was taken to facilitate participation.

*Data collection*

Interviews were selected as the mode of data collection to enable in-depth exploration of personal behaviours and build rapport to promote honest and candid disclosure. Interviews were offered as in-person at the point of recruitment or later, remotely, using secure video-conferencing software or by telephone. Sessions lasted between 10 and 40 minutes. A topic guide was used (see below) to explore their experiences of using the tracks, whether and to what extent installation of the tracks has changed their physical activity behaviour and levels, and factors that have contributed to their use of the tracks.

*Compensation*

Participants received a £10 shopping voucher following completion of the interview as a token of thanks.

**N.B.** All topic guides were pilot tested by the Applied Research Collaborations West Midlands lived experience panel and iteratively amended as the interviews/focus groups progressed.

**Evaluation of Every Step Matters track** **installations in Birmingham**

**Interview topic guide - Staff**

This is the initial topic guide. The overarching objectives will remain the same, but questions and prompts will be developed as interviews are undertaken to incorporate any important themes emerging from the interviews.

**TOPICS TO BE COVERED IN THE INTERVIEW**

1. What is your current job role?
2. How long have you been in your role?
3. What was your involvement in the development of the track programme?

*Prompts:*

- *How were the locations chosen?*
- *What aspects of safety were taken into consideration?*
- *How do the locations of the tracks relate to health inequalities?*
- *Did the plan for the tracks change during the course of the programme? In what way?*

1. What was your involvement with the installation of the tracks?

*Prompts:*

- *Were you involved in the development of the tracks? If so, how were you involved?*
- *Were you involved in the installation of one or more of the tracks? If so, how were you involved?*
- *Have you installed tracks in areas with different socio-economic characteristics? If so, was anything done differently to accommodate those likely to use the tracks?*
- *Have you installed other tracks outside of this project? If so, has this project been any different?*

1. Have you worked with BCC/Legacy WM before?

*Prompts:*

- *If so, how did this project differ?*
- *What was your experience of working with BCC/Legacy WM on this project?*

1. What problems/difficulties arose during the installations?

*Prompts:*

- *Problems with the development process? Problems with the installation process?*
- *Where/how did the problems arise? Communication? Other agencies? Admin problems?*

1. How did you go about resolving these problems?

*Prompts:*

- *Were they easy to resolve? Time consuming? Required additional input from other agencies/departments?*

1. What worked well and why?

*Prompt:*

- *Would you recommend using the same approach(s) for future projects?*

1. Are there any areas where improvements to the process could be made for future projects?

*Prompts:*

- *How could they be improved?*
- *What input would be required to implement this and by who?*

1. Were you involved in promoting the tracks? If so, how?

*Prompts:*

- *Advertising in community spaces*
- *Launch events, - what was involved?*
- *Were the community involved in the installations?*

1. Do you know anything about the use of the tracks since their installation?

*Prompts:*

- *How have they been used?*
- *Are you aware of any feedback about the tracks from the community? If so, what was the feedback?*
- *How has the use of the tracks changed since/during installation? (i.e. guided walks)*
- *Have these changes impacted costs and resources?*
- *What are the future-plans for the tracks beyond the scheme end date? I.e. will guided walks continue? Who will be responsible for the tracks?*

1. What are your views on the tracks?

*Prompts:*

- *What are the benefits of having a local track?*
- *What do you think the barriers are to people using the tracks and why?*
- *What do you think will encourage people to use the tracks and why?*

1. Is there anything you would change about the programme now it is nearing completion?

*Prompt:*

- *What would you do differently and why?*

1. Is there anything else you would like to add that we haven’t covered?

**End of interview –thank participant for their time and input.**

**Evaluation of every step matters track installations in Birmingham**

**Focus group/interview topic guide –non-users of the tracks**

This is the initial topic guide. The overarching objectives will remain the same, but questions and prompts will be developed as focus groups/interviews are undertaken to incorporate any important themes emerging from the focus groups/interviews.

**Consent will be taken prior to the date of the focus group/interview over the telephone and will be audio-recorded.**

**TOPICS TO BE COVERED IN THE FOCUS GROUP/INTERVIEW**

1. Please briefly introduce yourself to the other participants.

*Prompts: first name only, how long you have lived in the area.*

1. Do you have a current exercise routine?

*Prompts: What type of exercise do you do?*

*Where do you do your exercise (gym, home, etc)*

*How frequently do you exercise?*

1. Would you like to be more active?

*Prompts: How would you like to be more active?*

*What would help you to be more active?*

*Would you consider using the park for your exercise?*

1. Are you aware of the mile track that has been installed in your local park?
2. Would you consider using the track as part of your exercise routine?

*Prompts: Is the location of the track inconvenient for you?*

*Do you worry about your safety whilst using the track? If so, what is of particular concern?*

*Is the weather a concern for you?*

*For those who go to the gym would you consider doing exercise outside of this setting?*

1. In your opinion what do you think could be the benefits of using the track?

*Prompts: are there any health benefits associated with using the track?*

*Do you think there are any cost savings from using the track (no specialist equipment needed, no fees etc)?*

*Could using the track be more convenient and time saving?*

*Do you think there are any social benefits to exercising in the park?*

1. How do you think we can encourage more widespread use of the track?

*Prompts: What is the best way to approach people - Through community groups, social media, leaflets, individual personalised approach?*

*What form should this support take, practical, psychological, or social support?*

*Who should deliver the support and how?*

1. Is there anything else you would like to add that we haven’t covered?

**End of focus group/interview – thank participant for their time and input**

**Evaluation of every step matters track installations in Birmingham**

**Interview topic guide – Track users**

This is the initial topic guide. The overarching objectives will remain the same, but questions and prompts will be developed as interviews are undertaken to incorporate any important themes emerging from the interviews.

**TOPICS TO BE COVERED IN THE INTERVIEW**

1. How long have you lived in this area?
2. What exercise did you do before the installation of the track?

*Prompts: What types of exercise did you do?*

*Where did you exercise i.e. Gym, swimming pool?*

*How often did you exercise?*

1. How and when did you become aware of the track in your local park?

*Prompts: Leaflet through door, article in local paper, word of mouth, canvassing by Legacy West Midlands staff?*

1. How long have you been using the track for? i.e. since installation or more recently

*Prompts: Did you start using the track as soon as you were made aware of it?*

*Did you wait to see whether others used the track?*

1. Why did you start using the track as part of your exercise regime?

*Prompts: Encouragement from friends and family?*

*Convenience? Cost effectiveness? Ease of use?*

*Recommendation from a medical professional for health reasons?*

1. In your opinion what are the benefits of using the track?

*Prompts: Have you experienced any health benefits since using the track?*

*Have you noticed any cost savings from using the track (no specialist equipment needed, no fees etc)?*

*Is using the track more convenient and time saving compared to your previous exercise regime?*

1. What are the downsides of using the track?

*Prompts: Do you have any safety concerns about using the track?*

*Does the weather put you off using the track?*

1. How do you think we can encourage more widespread use of the track?

*Prompts: What is the best way to approach people? Through community groups, social media, leaflets, individual personalised approach?*

*What form should this support take practical, psychological, or social support?*

1. Is there anything else you would like to add that we haven’t covered?

**End of interview – thank participant for their time and input**
